# Intrauterine Device Migration Into the Rectum: A Case Report

**DOI:** 10.7759/cureus.83036

**Published:** 2025-04-26

**Authors:** Fatemah Alrowili, Lama J Albaish, Kawthar H Alabduljabbar

**Affiliations:** 1 Obstetrics and Gynecology, King Fahad University Hospital, Khobar, SAU; 2 College of Medicine, Imam Abdulrahman Bin Faisal University, Dammam, SAU

**Keywords:** case report, intrauterine device, migration, perforation, rectum

## Abstract

Intrauterine devices (IUDs) are an accepted and popular method of contraception for women looking for long-term pregnancy prevention. There are possible risks and complications, yet migration remains uncommon. Here, we report a case of IUD migration through the uterus into the cul-de-sac and the rectum. A 27-year-old female without any notable medical history had a history of a copper IUD insertion after she had delivered her second baby with the aim of contraception. Six months later, an unintended pregnancy occurred which was delivered successfully. After five years of insertion, she felt like a thread coming out of the anal opening upon defecation with on-and-off abdominal pain and constipation. During a pelvic examination, the IUD string was felt in the rectum. While imaging showed a retroverted uterus with an IUD seen in the left cul-de-sac space, the end of the IUD was seen directed and sited within the left lateral rectal lumen. The patient was referred under the care of the general surgery department electively for IUD removal as a case of anal fissure for lateral internal sphincterotomy. A suspected case of IUD migration should undergo gynecological analysis and radiological imaging. Finally, a prospective investigation and follow-up of the IUD after insertion are needed.

## Introduction

Intrauterine devices (IUDs), also called intrauterine contraception, are an accepted and popular method of contraception for women looking for long-term pregnancy prevention [1]. Universally, 14.3% of women of reproductive age use IUDs [2]. There are possible risks and complications, especially in the first few months of insertion, such as abnormal bleeding, dysmenorrhea, and unplanned pregnancy. Additionally, there is a risk of expulsion which can be up to 25%, while migration after intrauterine perforation is rare [3]. Nearly 1-3 of 1,000 IUD insertions lead to migration [4]. Although there are several directions of migration, the most common are into the peritoneal cavity and rarely into the surrounding pelvic organs, including the bladder, rectosigmoid, omentum, appendix, peritoneum, small intestine, adnexa, and iliac vein [3]. The World Health Organization (WHO) recommends surgical removal of a dislocated IUD regardless of its type [5]. Here, we report a case of an IUD migrating through the uterus into the cul-de-sac and the rectum.

## Case presentation

A 27-year-old female had no notable medical history (para: 3 + 3, all were normal vaginal deliveries). The patient had a history of a copper IUD insertion after eight months of delivering her second baby with the aim of contraception. The IUD was inserted uneventfully and, afterward, there was no follow-up to check the placement of the IUD. Six months later, an unplanned third pregnancy occurred, and she thought that the IUD fell on its own. Throughout her ultrasonographic follow-up for her baby, the IUD was not seen, and the pregnancy continued with the outcome of a healthy baby. Five years after the delivery of her third pregnancy, she felt like a thread coming out of the anal opening upon defecation with on-and-off abdominal pain and constipation. She assumed that it was the remnant of a suture from her last delivery.

The patient was followed up in a private hospital where she underwent a trans-abdominal ultrasound which failed to visualize the IUD in the uterine cavity. Subsequently, a plain abdominal X-ray identified the migration of the IUD, but the precise localization was hard to judge. After that, she was referred to King Fahad University Hospital. During the pelvic examination, the IUD string was felt in the rectum.

**Figure 1:**
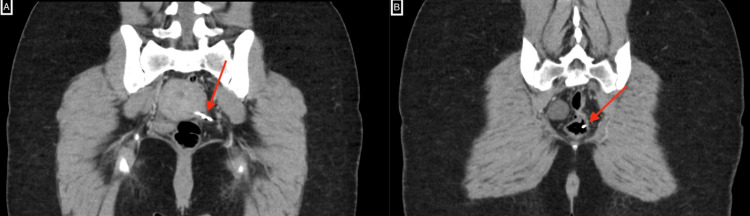
Contrast-enhanced CT of the pelvis showing the penetrating intrauterine device. The intrauterine device (red arrow) can be seen outside the endometrial cavity. Part of it can be seen penetrating the serosa of the uterus (A) and extending into the rectum (B).

The patient was then scheduled for a pelvic MRI with contrast which failed to detect the IUD. However, an incidental retroverted uterus was detected. Her pelvic organ and anal canal were unremarkable. Due to the high suspicion, a pelvic CT without contrast was obtained. An IUD was seen on the left side of the pelvis. The T (horizontal) part was seen in the left cul-de-sac space, with the right limb abutting and inseparable from the outer uterine wall. The left limb was abutting the left adnexa which otherwise appeared unremarkable. The end of the IUD was seen directed and sited within the left lateral rectal lumen. No collection or other abnormalities were seen in the pelvis. The endometrial cavity was empty. Some free fluid seen in the pelvis was likely physiologic. The final impression was a migrated IUD to the rectum (Figures 1A, 1B).

**Figure 2:**
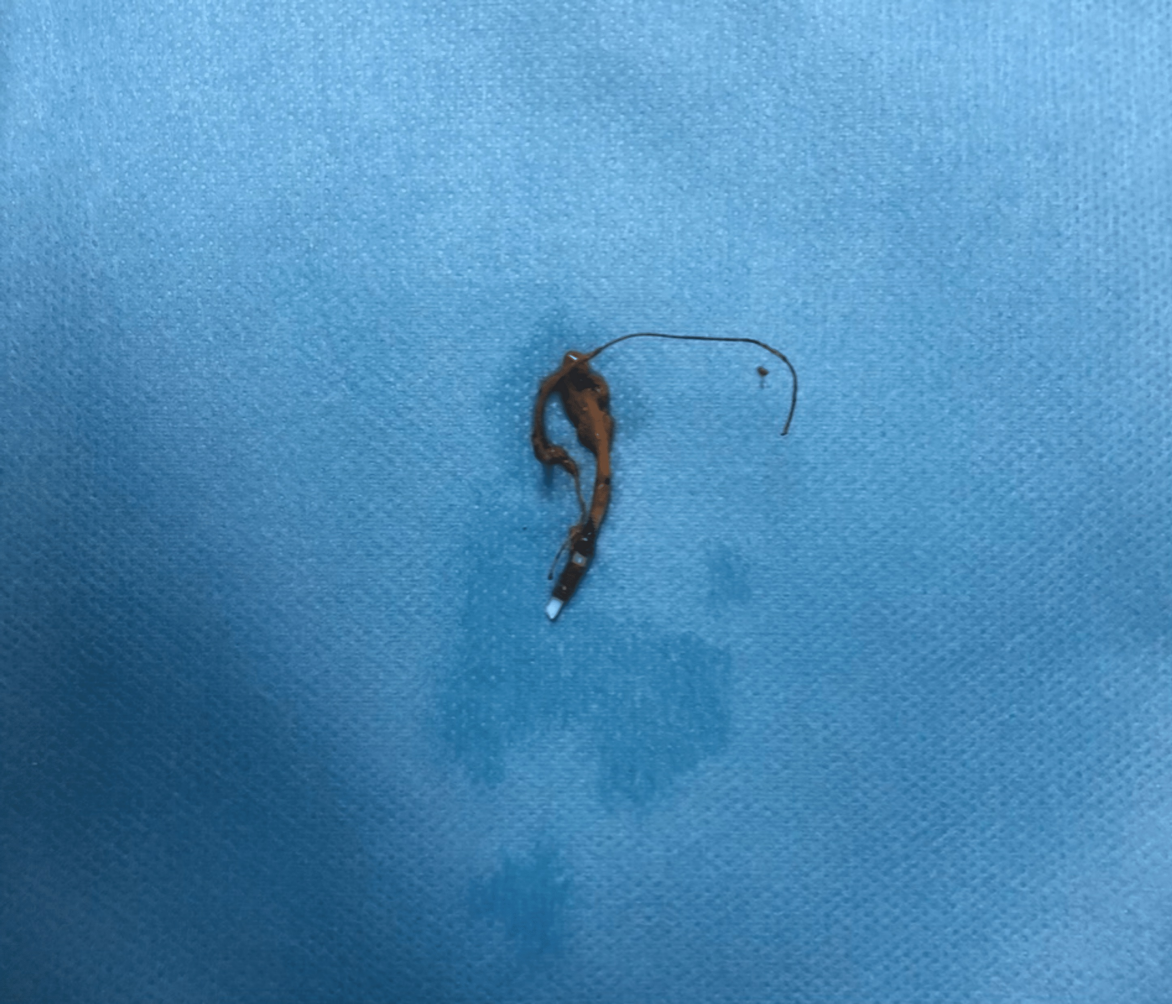
The retrieved intrauterine device.

The patient was referred under the care of the general surgery department electively for IUD removal as a case of anal fissure for lateral internal sphincterotomy. The IUD was removed under direct vision by slightly pulling on the string. Figure 2 shows the retrieved IUD.

The patient was followed up one week after the removal of the IUD in the general surgery outpatient clinic with good clinical improvement.

## Discussion

The IUD is one of the commonly used long-term contraceptive methods. Two types of IUDs are available and approved by the Food and Drug Administration (FDA), namely, copper and levonorgestrel IUDs (trade names: Mirena, Liletta, Skyla, and Kyleena), which vary in their dose and approved length of use [6]. Copper IUDs can be used for up to 10 years, while levonorgestrel IUDs can be used for up to three to five years, according to the FDA. IUDs are generally considered safe and effective; however, they can result in complications, including ectopic pregnancy, pelvic inflammatory disease, abdominal and pelvic pain, infection, uterine perforation, and expulsion [7]. However, when compared, levonorgestrel IUDs are associated with a lower risk of pregnancy and ectopic pregnancy [8]. Moreover, a study showed that perforations were more common with the levonorgestrel IUDs than with copper IUDs [5]. On the other hand, Jensen et al. did not find any uterine perforation in 500 levonorgestrel IUD insertions [9].

Uterus perforation by IUD is considered a serious yet uncommon complication, with the incidence ranging from 0.4 to 1.6 per 1,000 insertions [7]. There are two types of perforations, i.e., primary and secondary. Primary perforations occur during insertion and are associated with severe abdominal pain. Secondary perforation is a delayed event, suggested to be caused by gradual pressure necrosis of the uterine wall [3]. Uterine perforation is classified into complete perforation and partial perforation. In complete perforation, the IUD passes through all the uterine wall layers, including the endometrium, myometrium, and serosa, and can lie freely in the peritoneal cavity or migrate to another rare area. On the other hand, when the IUD gets fixed in the myometrium, it is referred to as partial perforation [10]. Furthermore, studies have shown that nulliparous patients have a higher expulsion rate compared to parous patients where the risk is 5-10% within five years after insertion and recurs in 30% of these patients [5].

Symptoms of IUD perforation vary depending on the site of migration [10]. Usually, bowel perforation is asymptomatic, with incidental discovery reaching up to 85% [3]. However, several symptoms might be experienced, such as abdominal pain, intermittent diarrhea, fever, and rectal bleeding. When the urinary tract is affected, suprapubic pain, dysuria, frequency, hematuria, and urinary tract infections can be experienced. In a previous case, a reported IUD resting on the lumbosacral plexus caused right-sided sciatica [10]. In our case, the patient presented with on-and-off abdominal pain and constipation.

The removal thread of the IUD can go missing which can be a warning sign, but it does not necessarily imply dislocation of the device. Moreover, the device can be incorrectly placed while the removal thread is visible which is why clinical diagnosis has a minor role in confirming the perforation. Other investigations must be conducted to confirm and localize the device. The best investigation that helps in precisely localizing a perforated IUD so far is imaging. Ultrasound is considered a bedrock investigation; however, it can help in identifying IUDs that are intrauterine rather than extrauterine. Furthermore, transvaginal scanning is better at localizing a perforated device than transabdominal scanning. Both types of IUD are radiopaque and are visible in a whole abdominal and pelvic X-ray, but the precise localization could be hard to judge. A CT scan or MRI can localize devices of any type [10]. In our case, the imaging showed a retroverted uterus with an IUD seen in the left cul-de-sac space, with the end of the IUD directed and sited within the left lateral rectal lumen.

According to the WHO, IUD removal should be done as soon as possible even if the patient is asymptomatic [3]. The recommended management is the surgical removal of the perforated IUD after localizing the device. This is usually done laparoscopically, a minimally invasive technique that allows for the retrieval of these devices in most cases. Other minimally invasive methods include hysteroscopy, cystoscopy, and colonoscopy, with the choice depending on the location of the perforated IUD [3,11]. In our case, direct visualization was done through the anal sphincter while the perforation was down the rectum by slightly pulling the device outside. No medical management was required after that.

## Conclusions

IUDs are popular and effective measures for contraception with multiple complications, yet migration remains rare. A comprehensive approach involving gynecological analysis, radiological imaging, and a collaborative general surgery team is an effective way to manage a suspected case of IUD migration, especially in cases where it has relocated to the rectum. Finally, a prospective investigation and follow-up of the IUD after insertion are needed.
